# Safety and efficacy of ARNI (valsartan/sacubitril) vs ACEI (enalapril) in acute heart failure – A prospective observational study

**DOI:** 10.1016/j.ihj.2022.04.003

**Published:** 2022-04-26

**Authors:** Tahir Saleem Bhat, Imran Hafeez, Sobia Fatima Tak, Asif Mattoo, Aamir Rashid Patigaroo, Abad Khan, Ajaz Ahmad Lone, Jahangir Rashid Beig

**Affiliations:** Sher-i-Kashmir Institute of Medical Sciences, Soura, Srinagar, Jammu and Kashmir, India

**Keywords:** Acute decompensated heart failure, Angiotensin converting enzyme inhibitor, Angiotensin receptor–neprilysin inhibitor, Heart failure, Valsartan/sacubitril

## Abstract

**Objective:**

To compare the safety and efficacy of valsartan/sacubitril (angiotensin receptor neprilysin inhibitor [ARNI]) against enalapril (angiotensin-converting enzyme inhibitor [ACEI]) in patients with acute heart failure at 6-month follow-up.

**Methods:**

In this prospective, single centre, and observational study conducted between September 2017 and February 2020 in India, patients with acute decompensated heart failure with reduced ejection fraction (<40%) were included. Patients were divided in two groups: valsartan/sacubitril (ARNI) group and enalapril (ACEI). Patients were followed up for at least 6 months after administration of first dose and were evaluated for safety, efficacy, and tolerability of target drug. Student's independent *t*-test was employed for comparing continuous variables. Chi-square test or Fisher's exact test, whichever appropriate, was applied for comparing categorical variables.

**Results:**

A total of 200 patients were included in the present study, 100 each in ARNI and ACEI group. The mean age of the population was 61.2 ± 8.4 years and 62.6 ± 8.6 years in ARNI group and ACEI group, respectively. The mean maximum tolerated dose by population in ARNI group was 203.6 mg and 8.9 mg in ACEI group. Readmission for heart failure were seen significantly higher in ACEI group than ARNI group (*p* value = 0.001). Parameters like ejection fraction, left ventricular end diastolic and systolic dimensions, 6 min walk test and Kansas City Cardiomyopathy Questionnaires (KCCQ) showed *p* values < 0.05 between the groups.

**Conclusion:**

The ARNI study group showed better safety and efficacy outcomes at the end of 6 months follow-up compared to ACEI group.

## 1 Introduction

Decrease in ability of heart to pump or fill the blood is known as heart failure. Heart failure is affecting more than 26 million population worldwide.^1^ Thus, it is termed as global pandemic. The mortality rates are also high and also decreases quality of life of affected population. Rapid onset of sign and symptoms of heart failure (HF) is termed as acute decompensated heart failure (ADHF). ADHF usually leads hospitalisation and can be fatal if urgent medication is not provided. Along with high morbidity and mortality, patients with HF also have greatly compromised health-related quality of life. The clinical presentation of ADHF usually ranges from moderate volume overload, congestion, low cardiac output and hypoperfusion with or without congestion.

Endogenous neurohormonal mechanisms like the renin-angiotension aldosterone system (RAAS), the sympathetic nervous system and the natriuretic peptide system are the prime reason for HF.^2^ The RAAS is responsible for retaining the sodium and water levels to maintain haemodynamic stability and also modulates vasoconstriction. When decrease in cardiac output is detected by sympathetic nervous system, it responds by increasing the adrenergic activity. The most recent target for HF treatment is the natriuretic peptide system.^3^ It maintains appropriate haemodynamics and plasma volume. Since last two decades, augmenting therapy with natriuretic peptide is considered as therapeutic strategy.^4^ However, oral administration of natriuretic peptide is ineffectual and long-term parenteral delivery is difficult for patient.^2^ Thus, Angiotensin receptor neprilysin inhibitors (ARNI), a new class of drug was developed to block both RAAS and augment natriuretic peptide.^5^^,^^6^ The present study was aimed to compare the safety and efficacy of valsartan/sacubitril (ARNI) against enalapril (angiotensin-converting enzyme inhibitor [ACEI]) in patients with acute heart failure at 6-month follow-up.

## 2 Methods

This was a prospective, single centre and observational study conducted between September 2017 and February 2020 in India. Patients diagnosed with acute heart failure with <40% left ventricular ejection fraction were eligible for inclusion. The inclusion criteria were: (1) age >18 years of either gender, (2) acute heart failure with reduced ejection fraction (<40%), (3) acceptable renal functional (glomerular filtration rate >30 ml/min/1.73 m^2^) and potassium level ≤5.0 mmol/l at the time of admission, (4) systolic blood pressure ≥100 mmHg at admission. The patients were excluded if: (1) history of hypersensitivity or allergy to any of the study drugs, as well as known or suspected contraindications to the study group, (2) history of angioedema, (3) acute coronary syndrome, (4) history of severe pulmonary disease, (5) symptomatic hypotension. The study protocol was approved by Institutional Ethics Committee and informed consent form was signed by each participating patient.

Patients who were in shock were stabilized with inotropes and vasopressors before enrolling in the study. After initial hemodynamic stabilization (off intravenous diuretics, inotropes and vasopressors for at least 24 h), patients were divided in two groups; valsartan/sacubitril (angiotensin receptor neprilysin inhibitor [ARNI]) group and enalapril (angiotensin-converting enzyme inhibitors [ACEI]). The ARNI group was started on valsartan/sacubitril from the lowest dose of 50 mg BD. The dose escalation was gradually done to maximum tolerated/maximum permissible dose of 200 mg BD. Similarly, in ACEI group, enalapril was started on 2.5 mg BD and uptitration of dose was done to maximum tolerated/maximum permissible dose of 10 mg BD. The uptitration was based on the treating physician's judgement.

Those who could not tolerate the target dose were downtitrated to lower dose at treating physician's discretion. The tolerability assessment was done in terms of hypotension, azotaemia, hyperkalaemia, angioedema. After administration of first dose, patients were kept under observation in the hospital for 72 h. Patients were followed up one week post discharge and then on 4th week, 12th week and 24th or 48th week. On each follow up, patients were assessed for safety, efficacy and tolerability of target drug. Safety of valsartan/sacubitril with enalapril in patients with acute heart failure was measured in terms of tolerability and adverse events (hypotension, azotemia, hyperkalemia, angioedema and other adverse events). The escalating strategy of the drug was decided by the treating physician on every visit based on the tolerability and side effect profile of the drug as deemed necessary by the physician.

The primary endpoint was safety and efficacy of the ARNI. It consisted of composite of cardiovascular death and rehospitalisation due to heart failure. The secondary endpoints were 6 min walk test, echo diameters and dimensions, hypotension and azotemia. Efficacy of valsartan/sacubitril was measured from the events of cardiovascular mortality and heart failure re-hospitalization over period of 6–12 months in comparison to enalapril in this patient population. Crossover to other group was allowed based on type and severity of adverse effect and as deemed appropriate by treating physician.

### 2.1 Statistical analysis

Continuous variables were expressed as mean ± standard deviation and categorical variables were summarized as frequencies and percentages. Student's independent *t*-test was employed for comparing continuous variables. Chi-square test or Fisher's exact test, whichever appropriate, was applied for comparing categorical variables. A *p*-value of less than 0.05 was considered statistically significant. All *p*-values were two tailed. Data were analysed in SPSS Version 22.0 (SPSS Inc., Chicago, Illinois, USA).

## 3 Results

A total of 200 patients were included in the present study; 100 each in ARNI group and ACEI group. The mean age of the population was 61.2 ± 8.4 years and 62.6 ± 8.6 years in ARNI group and ACEI group, respectively. Male population was predominant in both the groups; 70% and 74% in ARNI and ACEI groups, respectively. Smoking was the major risk factor reported 65% in ARNI group and 57% in ACEI group, followed by hypertension and diabetes mellitus. Left bundle branch block was seen in 15% and 13% patients in ACEI and ARNI group, respectively. There were 47% patients in ARNI group had NYHA class III heart failure while in ACEI group 42% had NYHA class III heart failure. The basic demography of population is given in Table 1.

**Table 1:** Basic demography of patients.

Parameters	ARNI (n = 100)	ACEI (n = 100)
Age (mean ± SD, y)	61.2 ± 8.4	62.6 ± 8.6
Male, *n* (%)	70 (70%)	74 (74%)
Hypertension, (%)	55%	47%
Diabetes mellitus, (%)	31%	33%
Smoker, (%)	65%	57%
History of CAD, (%)	11%	9%
Chronic kidney disease	2%	1%
Obesity	3%	2%
Hypothyroid	2%	4%
Chemotherapy	1%	0%
Smokers	65%	57%
SBP, (mean ± SD, mmHg)	107.6 ± 9.4	106.8 ± 10.8
DBP, (mean ± SD, mmHg)	65.3 ± 7.1	64.3 ± 7.6
Denovo heart failure, (%)	29%	25%
NYHA class II	36%	38%
NYHA class III	47%	42%
NYHA class IV	17%	20%

ARNI: angiotensin receptor neprilysin inhibitor; ACEI: angiotensin-converting enzyme inhibitors; SD: standard deviation; SBP: systolic blood pressure; DBP: diastolic blood pressure.

In ARNI group, patients were given drugs in range of 50 mg–400 mg. The mean maximum tolerated dose by population in ARNI group was 203.6 mg. Similarly, in ACEI group, patients were prescribed doses ranging from 2.5 mg OD to 10 mg BID. In ACEI group, the mean maximum tolerated dose was 8.9 mg. All the patients were followed up for minimum 6 months on maximum tolerated dose of ARNI or ACEI. Various parameters were measured at baseline and at 6-month follow-up are shown in Table 2. Many parameters like ejection fraction, left ventricular end diastolic and systolic dimensions, 6 min walk test and Kansas City Cardiomyopathy Questionnaires^7^ (KCCQ) showed *p* values < 0.05. Readmission for heart failure were seen significantly higher in ACEI group than ARNI group (*p* value = 0.001).

**Table 2:** Comparison of various parameters at baseline and at 6-month follow-up.

Parameters	ARNI		ACEI		*p* value
	At baseline	6-month	At baseline	6-month	
LVEF (mean ± SD, %)	34.1 ± 4.9	36.8 ± 4.4	32.9 ± 3.0	33.5 ± 3.1	<0.001
Left atrium (mean ± SD, mm)	38.3 ± 5.5	37.0 ± 4.4	38.4 ± 1.9	37.8 ± 2.2	0.98
LVEDD (mean ± SD, mm)	59.6 ± 4.4	56.9 ± 4.7	60.3 ± 3.1	59.4 ± 3.2	<0.001
LVESD (mean ± SD, mm)	47.3 ± 5.4	44.4 ± 5.4	47.5 ± 3.0	45.8 ± 3.0	0.022
6min walk test (mean ± SD, m)	152.8 ± 13.2	294.2 ± 102.5	145.7 ± 10.1	246.3 ± 15.1	<0.001
KCCQ (mean ± SD)	55.6 ± 3.8	77.8 ± 5.1	54.4 ± 3.9	72.2 ± 2.5	<0.001
Total readmission for HF, %	–	13	–	33	0.001

LVEF: left ventricular ejection fraction; LVEDD: left ventricular end diastolic dimension; LVESD: left ventricular end systolic dimension; KCCQ: Kansas City Cardiomyopathy Questionnaire; HF: heart failure.

Hypotension and general weakness were the major adverse events reported in ARNI group in 21% and 20% population, respectively. In ACEI group, renal impairment was reported in 13% population. The cardiovascular death related to heart failure were observed in 5% and 10% patients in ARNI and ACEI groups, respectively. The non-cardiac death was observed in 3 patients in ARNI group and in 2 patients in ACEI group. Two patients were lost to follow-up in ARNI group and one patient in ACEI group.

## 4 Discussion

In our study patients with acute HF with low ejection fraction (<40%) were treated with ARNI to determine its safety and efficacy in comparison with ACEI. Patients were followed up for at least 6 months. In the present study, male population was predominant in both the study groups. Similar trend was also reported in PIONEER HF^8^ study and TRANSITION^9^ study where male population was more than 70% of the total study population. However, some former studies did not report male dominancy, and reported <60% male population of the total study population.^10^, ^11^, ^12^, ^13^ The mean age of the population was 61.2 ± 8.4 and 62.6 ± 8.6 years in ARNI and ACEI group respectively. The mean age of population was more (>66 years) in TRANSITION study compared to the present study. A noteworthy factor in the present study was the amount of smoker population in both the group (65% and 57% in ARNI and ACEI, respectively). Other studies have shown, less number of smoker population (∼20%).^14^

Patients in both the groups were followed up for at least 6 months and various echocardiographic parameters showed significant difference between ARNI and ACEI group. Significant improvement was observed in LVEF in ARNI group than the ACEI group (*p* < 0.001). Similarly, left ventricular end diastolic dimension and left ventricular end systolic dimension decreased at 6-month follow-up in both the groups. The echocardiographic changes in the present study are comparable to the study performed by Almufleh et al.^15^ they further reported improvement in ejection fraction and reverse remodelling. In the present study, the mean dose administered in ARNI group was 203.6 mg, while Almufleh et al^15^ reported highest dose administered in ARNI group was 97–102 mg. The possible explanation for not achieving the target dose in majority of the patients was reluctance from the treating clinicians in enhancing the dose fearing the adverse effects of the drug, poor follow up, high cost of ARNI and poor compliance to drug. Distance covered in 6-min walk test also improved significantly in both the groups at 6-month follow-up. The improvement in the ARNI group was significantly higher than the ACEI group (*p* < 0.001). Health-related quality of life was also improved significantly in ARNI group compared to ACEI group (*p* < 0.001). The health-related quality of life improvement was determined by KCCQ and showed significant improvement in ARNI group compared to ACEI group (*p* < 0.001). A secondary analysis of PARADIGM-HF^16^ study also showed improvement in quality of life in terms of physical and social activity.

In the present study, the heart failure related death was reported in 5 patients in the ARNI group and in 10 patients in ACEI group. The major adverse event was hypotension reported in 21 patients in ARNI group while same was reported in 11 patients in ACEI group. Other than that, general weakness and giddiness were reported as adverse events in both the groups. Eight patients in the ARNI group and six patients in the ACEI group underwent cardiac resynchronization therapy after the completion of study period. To minimize bias, echocardiographies were performed by an independent operator who was unaware of patients' enrolment in the study, which added greater value to the study.

## 5 Conclusion

The ARNI study group showed better safety and efficacy outcomes at the end of 6 months follow-up in terms of death, echocardiographic parameters, readmission rate due to HF, 6 min walk test compared to ACEI group. The KCCQ showed improvement in both the groups, however ARNI group had significant increased compared to ACEI group.
